# Comparative genome analyses of mycobacteria give better insights into their evolution

**DOI:** 10.1371/journal.pone.0172831

**Published:** 2017-03-14

**Authors:** Wei Yee Wee, Avirup Dutta, Siew Woh Choo

**Affiliations:** 1 Genome Informatics Research Laboratory, Centre for Research in Biotechnology and Agriculture (CEBAR), High Impact Research Building, University of Malaya, Kuala Lumpur, Malaysia; 2 Department of Biological Sciences, Xi'an Jiaotong-Liverpool University, Suzhou Dushu Lake Science and Education Innovation District, Suzhou Industrial Park, Suzhou, P. R. China; Indian Institute of Technology Delhi, INDIA

## Abstract

Mycobacteria a genus of Actinobacteria are widespread in nature ranging from soil-dwelling saprophytes to human and animal pathogens. The rate of growth has been a classifying factor for the *Mycobacterium* spp., dividing them into the rapid growers and the slow growers. Here we have performed a comparative genome study of mycobacterial species in order to get better understanding of their evolution, particularly to understand the distinction between the rapid and slow growers. Our study shows that the slow growers had generally gained and lost more genes compared to the rapid growers. The slow growers might haved eventually lost genes (*LivFGMH* operon, *shaACDEFG* genes and MspA porin) that could contribute to the slow growth rate of the slow growers. The genes gained and lost in mycobacteria had eventually helped these bacteria to adapt to different environments and have led to the evolution of the present day rapid and slow growers. Our results also show high number of *Mycobacterium abscessus* specific genes (811 genes) and some of them are associated with the known bacterial quorum sensing genes that might be important for *Mycobacterium abscessus* to adapt and survive in variety of unfavorable environments. *Mycobacterium abscessus* also does not contains genes involved in the bacterial defense system and together with the quorum sensing genes may have contributed to the high gene gain rate of *Mycobacterium abscessus*.

## Introduction

Mycobacteria are a genus of Actinobacteria of the *Mycobacteriacae* family which are obligate aerobic, immobile, acid-fast Gram-positive bacteria with high G+C content, and are widespread in nature ranging from soil-dwelling saprophytes to pathogens of humans and animals [1,2]. *Mycobacterium* have been classified into two distinct categories depending on their rate of growth; the slow growers which take more than 7 days on subculture to form colonies, and then there are the rapid growers forming colonies within 3–7 days [3]. Most of the major pathogenic Mycobacteria such as *M*. *tuberculosis* and *M*. *leprae* responsible for causing serious diseases in humans and animals, such as tuberculosis and leprosy respectively, are slow growers. With regard to pathogenicity, some of the non-tuberculous mycobacteria (NTM) have also been shown to cause disease in humans especially in immunocompromised individuals [4]. However not all pathogenic NTMs are slow growers, such as *M*. *abscessus* a commonly isolated rapidly growing NTM is the third most common cause of lung disease [5]. It is believed that the slow growers have evolved from the rapid growing mycobacteria [6,7]. Interestingly, studies have also shown that a distinction between the rapid and the slow growers is in the presence of ESX-5, which are exclusively present in the slow-growing mycobacteria [8].

Here we present a pan-genome analysis of 28 mycobacterial species (comprised of both rapid and slow growers). This study was performed with the objective to have a better understanding of the distinction between the rapid and the slow growers at the genomic level of the evolution of the mycobacterial genus.

## Materials and methods

### Pan-genome analysis of *Mycobacterium* genus

28 genomes from different *Mycobacterium* species were downloaded from the NCBI database for comparative and pan-genome analysis (Table 1). For consistency, the 28 genomes were re-annotated using the Rapid Annotation using Subsystem Technology (RAST) pipeline [9]. The predicted protein sequences of the 28 strains from RAST were used pan-genome analysis using PGAP (pan-genome analysis pipeline) [10]. Functional orthologs among the 28 strains were searched using the Gene Family (GF) method [10]. The protein sequences of each strain were mixed together and marked with the strain identifiers. BLASTALL was first performed among the protein sequences with the minimum score value of 50 and e-value of 1e-8 [11,12]. The filtered BLAST results were clustered by MCL algorithm [13]. In order to group the same genes into clusters, the threshold for the global match region was set to a minimum of 50% of the longer gene protein sequence and 50% sequence identity.

**Table 1:** Details of 28 different *Mycobacterium* species used in the analysis.

*Mycobacterium* species	Strain Name	Genome size (Mbp)	GC %	Contig	ORFs
*Mycobacterium africanum*	GM041182	4.39	65.6	1	4,369
*Mycobacterium avium*	K10	4.83	69.3	1	4,643
*Mycobacterium chubuense*	NBB4	5.58	68.7	1	5,346
*Mycobacterium colombiense*	CECT3035	5.58	68.1	17	5,341
*Mycobacterium fortuitum*	DSM46621	6.35	66.2	82	6,135
*Mycobacterium gilvum*	Spyr1	5.55	67.9	1	5,393
*Mycobacterium hassiacum*	DSM44199	5.0	69.5	169	4,849
*Mycobacterium indicus pranii*	MTCC9560	5.59	68	1	5,326
*Mycobacterium intracellulare*	ATCC13950	5.4	68.1	1	5,148
*Mycobacterium kansasii*	ATCC12478	6.42	66.1	108	6,007
*Mycobacterium leprae*	TN	3.27	57.8	1	1,605
*Mycobacterium mageritense*	JR2009	6.5	66.4	1.031	6,313
*Mycobacterium marinum*	M	6.64	65.7	1	5,826
*Mycobacterium parascrofulaceum*	ATCC_BAA614	6.56	68.4	124	6,093
*Mycobacterium phlei*	RIVM601174	5.68	69.2	102	5,622
*Mycobacterium rhodesiae*	NBB3	6.42	65.5	1	6,426
*Mycobacterium smegmatis*	JS623	6.46	65.1	1	6,591
*Mycobacterium* sp.	JLS	6.05	68.4	1	5,933
*Mycobacterium* sp.	KMS	5.74	68.4	1	5,605
*Mycobacterium* sp.	MCS	5.71	68.5	1	5,558
*Mycobacterium brisbanense*	UM_WWY	7.69	66.4	130	7,590
*Mycobacterium thermoresistibile*	ATCC19527	4.87	69	56	4,683
*Mycobacterium tuberculosis*	CCDC5079	4.4	65.6	1	4,553
*Mycobacterium ulcerans*	Agy99	5.63	65.5	1	5,566
*Mycobacterium vaccae*	ATCC25954	6.25	68.6	33	5,966
*Mycobacterium vanbaalenii*	PYR1	6.49	67.8	1	6,291
*Mycobacterium abscessus*	ATCC19977	5.0	64.1	1	5,004
*Mycobacterium xenopi*	RIVM700367	4.43	66.1	117	4,427

The relation between pan-genome size and genome number was determined using the method described by Zhao et al. [14]. According to Tettelin’s review on pan-genome research [15], Heaps’ Law model is employed to fit the pan-genome size of strains. Thus, pan-genome size model:

$$y=A_{1}x^{B_{1}}+C_{1}$$

x denoting genome number, y denoting pan-genome size, *A*_1_, *B*_1_ and *C*_1_ the fitting parameters.

Core genome size model:

$$y=A_{2}e^{B_{2}x}+C_{1}$$

x denoting genome number, y denoting core genome size, *A*_*2*_, *B*_*2*_ and *C*_*2*_ the fitting parameters.

### Phylogenetic inferences

The phylogenetic trees of *Mycobacterium* isolates were first constructed using the identified core genes following the approaches described by Rokas and colleagues [16]. The protein sequences of these core genes were concatenated to construct a phylogenetic tree using Maximum Parsimony and 1000 times bootstrap resampling approach. Each of the concatenated sequences was aligned with ClustalW [17].

### Gene gain and loss detection

The constructed tree was used as a map for the evolution of each isolate and the insertion and deletion of genes were mapped onto the phylogenies according to the Maximum Likelihood principle. The evolution of the *Mycobacterium* was modelled by the gain and death (GD) and gain and death with the free rates model (GD-FR-ML) where each branch contains separate gene gain and death turnover rate [18].

### Identification of bacteria defense system genes

From the RAST genome annotation, bacterial defense system related genes such as the restriction modification (RM) system and the toxin-antitoxin (TA) system were identified in the *Mycobacterium abscessus* ATCC19977, *Mycobacterium tuberculosis* CCDC5079, *Mycobacterium africanum* GM041182 and *Salmonella* Paratyphi A.

The CRISPR in the mentioned bacteria was further predicted using CRISPRfinder [19].

## Results and discussion

28 different mycobacterial species comprising 16 rapid growing and 12 slow growing mycobacterial species were used in this study. For consistency, all 28 mycobacterial genomes were annotated using the RAST pipeline. The annotations of each genome are summarized in Table 1.

Overall, the genome size of the 28 mycobacterial strains ranged from 4Mbp to 6Mbp except for *Mycobacterium leprae* TN (around 3Mbp) which might due to genome degradation and *Mycobacterium brisbanense* UM_WWY which was the only mycobacterial genome having a genome size larger than 7Mbp.

### Comparative genomic analysis among *Mycobacterium* species

#### *Mycobacterium* pan-genome analysis

**Fig 1:**
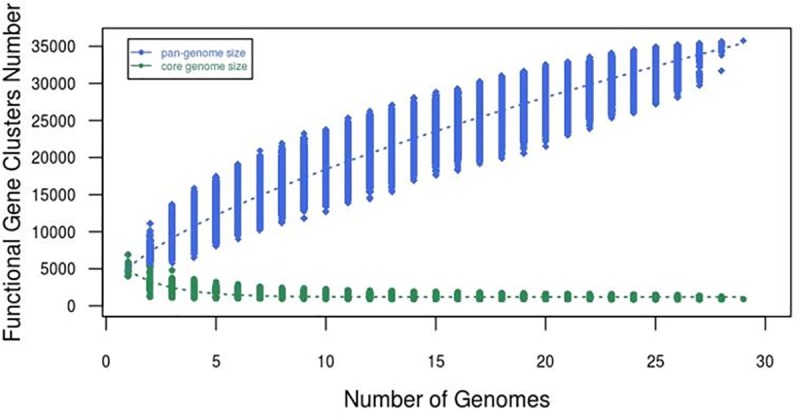
Prediction of *Mycobacterium* pan- and core- genomes size.

Using the predicted protein sequences of the 28 annotated *Mycobacterium* genomes as the input, the pan-genome analysis was conducted. Pan-genome analysis showed that the *Mycobacterium* genus had an open pan-genome suggesting that its pan-genome size of *Mycobacterium* could continue to increase when the number of genome added increased according to the following predicted functional model (Fig 1):

$$y=3806.24083816781^{\;*}x^{\mathrm{**}}0.651+1366.97772893166$$

y = Pan-genome size

x = Number of genome

In other words, this genus is expected to continue to gain genes and evolve in the future. This may be also explained by the high number of accessory gene clusters (95%) that we observed in this genus. A total of 35,751 unique gene families were identified from the 28 mycobacterial genomes which comprised of 1,829 (5%) core gene clusters and 33,922 (95%) accessory gene clusters. Of the 33,922 accessory genes families, 13,370 are species-specific genes which accounted around 40% of the accessory genes.

### *Mycobacterium* core-genes derived phylogenetic tree

**Fig 2:**
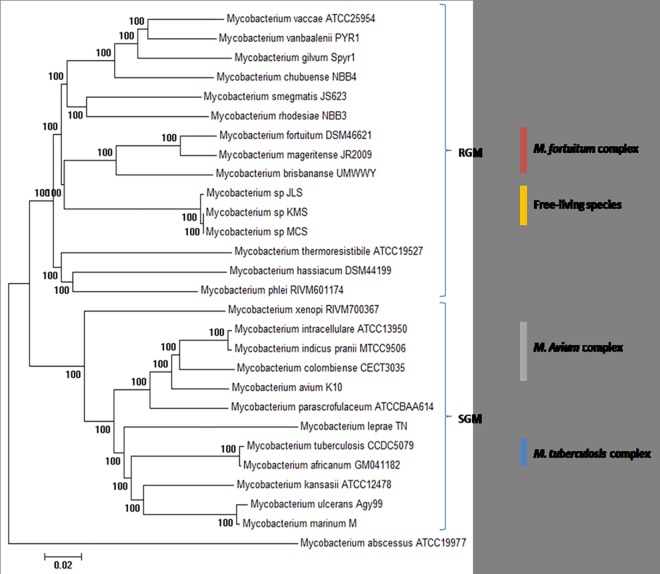
Phylogenetic tree of *Mycobacterium* species constructed using 531 core-concatenated genes. Bootstrap numbers were generated in 1,000 runs and nodes with bootstrap support values of 100 were indicated.

The 16S rRNA gene has been used for years as the candidate gene for phylogenetic studies for Mycobacterium [20]. However, it has been reported that this housekeeping gene might not produce a robust phylogenetic tree for mycobacteria due to the limited interspecies genetic variability in this genus [7]. Hence, the concept of concatenation of multiple housekeeping genes was proposed for generating a more robust phylogenetic tree [21]. To infer the phylogenetic relationship of *Mycobacterium* spp., we constructed a phylogenetic tree of 28 *Mycobacterium* spp. using the concatenated sequence of 531 core proteins with 1:1 orthologs found from the pan-genome analysis. The core genes were concatenated into a single 191,752 nucleotide alignment (Fig 2). As anticipated, the tree was in concordance with the known relationships for *Mycobacterium* spp. [22].

Interestingly, the generated tree clearly separated the rapid-growing mycobacteria (RGM) and slow-growing mycobacteria (SGM). For instance, the known slow growers, the *Mycobacterium tuberculosis* complex (*Mycobacterium tuberculosis* and *Mycobacterium africanum*), *Mycobacterium leprae*, *Mycobacterium ulcerans*, *Mycobacterium marinum* and the *Mycobacterium avium* complex species (*Mycobacterium avium*, *Mycobacterium colombiense*, *Mycobacterium intracellulare*) were clustered together. For the known rapid-growers such as *Mycobacterium vaccae*, *Mycobacterium vanbaalenii*, *Mycobacterium gilvum*, *Mycobacterium smegmatis*, *Mycobacterium fortuitum* complex members (*Mycobacterium fortuitum*, *Mycobacterium mageritense*, and *Mycobacterium brisbananse*) were clustered into a large clade and clearly separated from other rapid growing mycobacteria, as well as the free-living mycobacterial strains such as *Mycobacterium* sp. JLS, *Mycobacterium* sp. KMS and *Mycobacterium* sp. MCS. The results were also in concordance with the genus tree from a previous study which also generated based on the concatenation of core genes [18].

Interestingly, most of the SGM with few exceptions, appeared to contain smaller genome size compared to the RGM. Most of the SGM genome size was lesser than 6Mbp except for *Mycobacterium kansasii* which has a genome size of 6.42Mbp. On the other hand, RGM has genome size larger than 5.6Mbp except for *Mycobacterium gilvum*, *Mycobacterium chubuense*, *Mycobacterium hassiacum*, *Mycobacterium thermoresistibile* and *Mycobacterium abscessus*.

Besides, we also noticed high number of species-specific genes in the *Mycobacterium* genus which accounted for around 40% of the total pan-genome. The high number of species-specific genes indicated that horizontal gene transfer had played a major part in the evolution of *Mycobacterium* genus as many non-mycobacterial genes were brought into the *Mycobacterium* genomes [16]. *Mycobacterium brisbananse* UM_WWY contained the highest species-specific genes followed by *Mycobacterium abscessus* and *Mycobacterium smegmatis* while *Mycobacterium* sp. KMS and *Mycobacterium* sp. MCS had the lowest species-specific gene clusters.

From the result, we also observed that *Mycobacterium brisbananse* UM_WWY contains the highest species-specific gene clusters (1,631), followed by *Mycobacterium abscessus* 1,423 genes, *Mycobacterium smegmatis* 923 genes and *Mycobacterium kansasii* 683 genes. While *Mycobacterium* sp. KMS (25 genes) and *Mycobacterium* sp. MCS (26 genes) which contains the lowest species specific gene clusters. The well-known *Mycobacterium* species–*Mycobacterium tuberculosis* also contains a low number of species-specific gene clusters of 93 genes. However, the result here may not imply the true number of species-specific genes as only one strain from each species was included in the analysis. Minor part of the genes may be strain-specific gene rather than the species-specific genes. The variation of bacteria genome size may underlie many important morphological, physiological, and behavioral differences between species and contribute much of the genetic and genomic diversity observed in nature [19,23–26].

The highest number of species-specific genes in *Mycobacterium brisbananse* UM_WWY was probably due to the high genome size of 7.69Mbp, the largest genome size among the *Mycobacterium* species. *Mycobacterium smegmatis* and *Mycobacterium kansasii* also contained genome sizes larger than 6Mbp. However, *Mycobacterium abscessus* having the second highest species-specific genes has a genome size of around 5Mbp only.

### *Mycobacterium* complexes and their evolution

**Fig 3:**
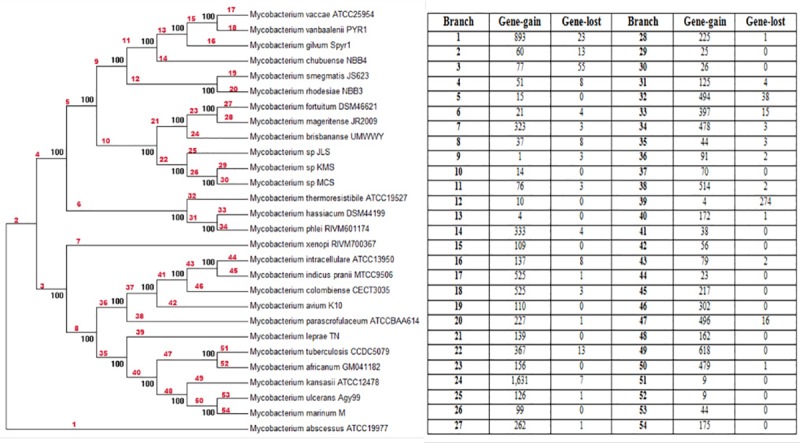
The recombination rate and number of gene gain and lost during the evolution of the *Mycobacterium* species.

From phylogenetic analysis, we observed that a few known *Mycobacterium* complexes such as *Mycobacterium tuberculosis* complex, *Mycobacterium avium* complex and *Mycobacterium fortuitum* complex were clearly grouped in the *Mycobacterium* genus tree and each complex had its own characteristics. The recombination rate and number of genes gained and lost has been shown in Fig 3. To investigate the recombination rate at these complexes, we examined the gene loss and gain events at the branch 21 for *Mycobacterium fortuitum* complex, branch 37 for *Mycobacterium avium* complex and the branch 47 for the *Mycobacterium tuberculosis* complex.

During the evolution of the *Mycobacterium fortuitum* complex, 139 genes were gained in this complex. For the *Mycobacterium avium* complex, 70 genes were gained, whereas 496 genes were gained (as well as 16 genes were lost) in the *Mycobacterium tuberculosis* complex. Interestingly, of the 496 gene gains in the *Mycobacterium tuberculosis* complex, we have found multiple genes involved in toxin-antitoxin (TA) systems. For instance, there was a pair of YdcD and YdcE toxin-antitoxin, Doc and Phd toxin-antitoxin and 10 toxins VapC with PIN domain. TA system is able to facilitate the bacterial adjustment in response to the environmental changes such as less stable conditions which may cause growth inhibition and eventually cell death. When the conditions become more favorable and stable, reactivation of the TA system may facilitate the bacterial survival [27]. Furthermore, the TA systems are also involved in defense mechanism such as anti-addiction against similar plasmid-borne activation, stabilization of genomic region and defense against the phage infection. Therefore, the gain of these defense systems in the *Mycobacterium tuberculosis* complex may suggest that *Mycobacterium tuberculosis* complex members could have more conserved genomic structure and probably are more resistant to the invasion of foreign DNA, for example, through the horizontal gene transfer (HGT). This may be further supported by the observations that the low number of genes gained at the branches 51 and 52 (which are the genome recombination rate) for *Mycobacterium tuberculosis* and *Mycobacterium africanum*. These findings might also explain why the genome size of the *Mycobacterium tuberculosis* complex members are generally the smaller (except for *Mycobacterium leprae* that might have undergone genome degradation and *Mycobacterium vaccae* ATCC25954 which is not a complete genome) and all below 4.5Mbp compared to other known *Mycobacterium* spp. that are not in the complex.

### Rapid and slow growing mycobacteria

From the generated core-genes phylogenetic tree, the mycobacterial species were clearly separated into two major clusters: rapid growers and slow growers except for *Mycobacterium abscessus* a rapid grower but emerged out as an outgroup. This was probably due to the high evolutionary rate in *Mycobacterium abscessus* which has already been shown to possess a high potential of gaining genes from the pan-genome analysis.

*Mycobacterium* genus may be differentiated into two classes at branch 3. Thus, branch 3 which is the recombination level for the slow growers may be responsible for the reason behind the differentiation of these 2 *Mycobacterium* classes. The results showed that during the mycobacterial evolution, all the slow growers had gained 77 genes and lost 55 genes. While on the other hand, the rapid growers gained 51 genes and lost 8 genes. According to Devulder, current mycobacterial species might have originated from ancestral rapid growing mycobacteria [7]. We believe that the slow-growers might have gained or losses some of the genes in order to evolve into the current SGM.

Interestingly, among the 55 genes lost in the slow growing mycobacteria, we found genes responsible for the access to extracellular nutrients which are related to the growth rate of mycobacteria. For instance, we found the slow-growers had lost a cluster of genes, the *LivFGMH* operon which encodes for the branched chain amino-acid transport ATP-binding LIV proteins. The LIV transport system has been reported to allow the transportation of leucine, isoleucine and valine into the bacteria which can enhance the growth of *Bacillus thuringiensis* [28]. The loss of these genes in the SGM might slow down the growth of these bacteria.

Besides, there was the cluster with 6 genes (*shaACDEFG*) found to be present in all the rapid growers but missing from the SGM species. They encode for a multi subunit transport system named as Sha, a Na^+^/H^+^ antiporter. The Sha system is a complex with cluster of 6 or 7 genes and is important for the homeostasis of Na^+^ and H^+^ under extreme condition [29]. Although, currently there are no evidences to indicate that the Sha system is responsible for the fast growth rate of the rapid growing *Mycobacterium*, but the Sha system allows fast growing *Mycobacterium* to adapt to different environmental changes and conditions which maybe the main reason that allowed rapid growers to have fast growth rate.

Besides that, the slow growers also lost a few genes which encode for the MspA porin protein. It has been reported that the low permeability of porin in slow growers may limit the efficiency of the hydrophilic drug in the bacteria and also growth rate of mycobacterium due to limited uptake of the polar nutrients [30]. Mailaender and colleagues have shown that the MspA porin is able to accelerate the growth of RGM [31]. The *mspA* gene has been extracted from *Mycobacterium smegmatis* and inserted into *Mycobacterium bovis* BCG and transformed with the expression vector. Their results showed that the *mspA*-expressing *Mycobacterium bovis* BCG strain grew significantly faster than the normal *Mycobacterium bovis* strain. The generation times were 25 hours for the *Mycobacterium bovis* BCG strain but 27 hours for the wild type strain [31]. Therefore, the loss of some genes in slow growers throughout the evolutionary time might be a main reason why these bacteria have become SGM.

Another interesting observation was, the slow growers showed the presence of components of Type VII secretion system which were absent in the rapid growers. All the slow growers had Type VII secretion AAA-ATPase EccA, Type VII secretion protein EccE and ESX-3 secretion system protein EccE3. It has been reported that type VII secretion systems have been used by *Mycobacteria* to secrete proteins across their complex cell envelope, while some of them have been shown to be essential for mycobacterial virulence and/or viability in pathogenic mycobacteria [6]. Recent studies have also shown that plasmids carrying ESX have played an important part serving as accelerators of adaptation and biodiversity with probable impact on the emergence of mycobacterial pathogenicity [32].

### *Mycobacterium abscessus*-specific genes

As mentioned earlier, the high number of species-specific genes did not necessarily indicate a larger genome size, as in the case of *Mycobacterium abscessus* which had the second highest species-specific genes but with a genome size of around 5Mbp only. The high number of species-specific genes for *Mycobacterium abscessus* led us to further study the function of the *Mycobacterium abscessus*-species specific genes and its defense system which may provide some interesting unique features of *Mycobacterium abscessus*.

**Fig 4:**
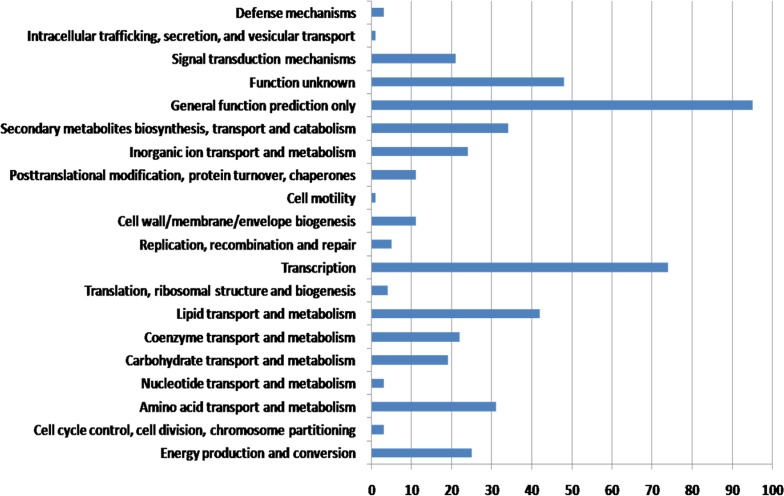
COG classification of *Mycobacterium abscessus*-specific genes.

To study the genetic differences between *Mycobacterium abscessus* and other mycobacterial species, we further reduced the species-specific number by comparing the current set of 1,423 genes with the genes from other 41 *Mycobacterium abscessus* strains. By comparing more *Mycobacterium abscessus* genomes, we were able to filter out genes which may be strain-specific gene rather than the species-specific genes. Results showed that 811 genes families were present in *Mycobacterium abscessus*, but not in other mycobacterial species. To examine the functions of *Mycobacterium abscessus*-specific genes, we performed a homolog search on the COG database on all the 811 genes (Fig 4). The *Mycobacterium abscessus*-specific genes showed the highest distribution in the general function classes (95 genes) followed by transcription (74 genes) and function unknown (48 genes). The function of the 421 *Mycobacterium abscessus*-specific genes (52%) remains unknown as they had been annotated as hypothetical proteins. Thus, more studies are needed to investigate the functions of these genes. Under the transcription category, there were 32 transcriptional regulator genes. The high number of transcriptional regulator genes specific to *Mycobacterium abscessus* may help in the survival of *Mycobacterium abscessus* in unstable environment as adaptive responses as mediated by transcriptional regulators [33].

Furthermore, out of the 811 gene families there were genes that were related to the bacterial quorum sensing. It has been reported that quorum sensing can cause virulence in bacteria by response to variety of signaling molecules called autoinducers [34]. This would allow bacteria to control their own gene expression and distinguish between low and high cell population density, which is important for them to adapt to environmental changes and also the changes in cell number [34]. Therefore, the acquisition of these *Mycobacterium abscessus*-specific quorum sensing genes/operons might be important to help *Mycobacterium abscessus* to survive in a wide range of adverse environmental conditions. One of the quorum sensing gene operons was the LuxI/LuxR signal-response system. It has been reported that this system controls the bioluminescence in *Vibrio fischeri* by monitoring the concentration acylated homoserine lactone (HSL), an autoinducer that regulates density-dependent light production in *Vibrio fisheri*. Moreover, researchers found that the homologs of LuxI and LuxR are also able to regulate the process of exoenzyme synthesis, conjugation, antibiotic production, luminescence and biofilm formation [35].

We also found a NTD operon consisting of *ntdA*, *ntdB* and *ntdC* (as well as a *glcP* gene) that were specific to *Mycobacterium abscessus*. The *glcP* located downstream of the *ntdABC* operon has been reported to co-express with the *ntdABC* operon [36]. The NTD operon is able to produce neotrehalosadiamine (NTD), amino acid antibiotics that act as an autoinducer. Besides that, the production of NTD can act as antibiotics which inhibit the growth of the other competing organisms [36].

Another *Mycobacterium abscessus*-specific quorum sensing operon was the phenazine operon. The secondary metabolites are usually produced by variety of bacteria especially pseudomonads. *Mycobacterium abscessus* possess 6 Phz homologues with three of the homologues (*phzC*, *phzD* and *phzE*) are linked and the organization of the genes are similar with *Pseudomonas aeruginosa*. Interestingly, it has been reported that the phz operon has a common ancestry and moved between species and inserted into *Mycobacterium abscessus* [37]. Phenazine is related to pathogenesis mainly due to their ability to generate reactive oxygen species (ROS) in other organisms and tissues. Bacterial virulence could be enhanced when phenazine production interfere the host cell functions. Thus, *Mycobacterium abscessus* might have gained some non-mycobacterial virulence genes and quorum sensing genes, further indicating that *Mycobacterium abscessus* has the capability to evolve and gain virulence genes.

### *Mycobacterium abscessus* lacks of genes in bacteria defense system

According to Makarova, prokaryotes defense systems can be categorized into 2 groups. The first involve defense system based on the self-nonself discrimination principle and second is based on the programmed cell death induced by infection. The first group of the defense system includes restriction modification (RM) system and the CRISPR (Clustered Regularly Interspaced Short Palindromic Repeats). RM system will attack non-self-invaders and CRISPR can memorize the foreign infectious agent and attack it afterwards. The second group of the defense system involves the toxin-antitoxin (TA) system [26,38]. With that in mind we searched for these bacterial defense system components in *Mycobacterium abscessus* and compared them to the other bacteria like *Mycobacterium tuberculosis*, *Mycobacterium africanum* and *Salmonella* Paratyphi A (Table 2).

**Table 2:** Bacterial defense system for *Mycobacterium abscessus* compared to other bacterial genomes.

Strain	RM system	TA system	CRISPR
*Mycobacterium abscessus* ATCC19977	0	0	0
*Mycobacterium tuberculosis* CCDC5079	1	13	8
*Mycobacterium africanum* GM041182	1	12	7
*Salmonella* Paratyphi A ATCC9150	2	1	3

Interestingly, we found no complete systems or operons encoding for the defense system in the *Mycobacterium abscessus* ATCC19977. There were no complete set of genes fully encoding the RM, TA systems in *Mycobacterium abscessus* genome. There were also no CRISPR in *Mycobacterium abscessus* from the prediction of the CRISPRfinder. On the other hand, *Mycobacterium tuberculosis* CCDC5079, *Mycobacterium africanum* GM041182 and *Salmonella* Paratyphi A ATCC9150 had a more conserved genomic structure with a number of genes involved in the defense system. *Mycobacterium tuberculosis* CCDC5079 had 1 complete RM system, 13 pairs of TA system and 8 predicted CRISPRs; *Mycobacterium africanum* GM041182 had 1 complete RM system, 12 pairs of TA and 7 predicted CRISPRs. Other than the *Mycobacterium* species, *Salmonella* Paratyphi A which has been shown contain conserved genome structure also contained these defense genes. Thus, the lack of genes involved in the defense system might cause *Mycobacterium abscessus* to be unable to recognize foreign DNA genome and allow genome insertion. Besides, quorum sensing genes enable *Mycobacterium abscessus* to have greater opportunity to get exposed to different harsh environments, which in turn may further enhance the possibility of insertion of the foreign materials.

## Conclusion

Our analysis suggests that the mycobacterial genus has undergone a series of gene gain and gene loss events in the course of its evolution which likely enable them adapt to different environments and led to the evolution of the present day rapid and slow growers.

## Supporting information

S1 Table Gene annotation of core genes and specific genes for the 28 *Mycobacterium* species. (XLSX)
